# Supplementary material for: Constructing a Shared Mental Model for Feedback Conversations: Faculty Workshop Using Video Vignettes Developed by Residents
Source: MedEdPORTAL. 2019 May 1;15:10821. doi: 10.15766/mep_2374-8265.10821 (PMC6519682; doi:10.15766/mep_2374-8265.10821)
Supplement: Supplementary file 1 — A. Facilitator Guide.docx B. Vignette Scripts.docx C. Cocky Connor.mp4 D. Constructive Conversation.mp4 E. Defensive Debbie.mp4 F. Distracted Attending.mp4 G. Impersonal Attending.mp4 H. Self-Effacing Sammy.mp4 I. Session Evaluation.docx J. Dimensions and Items.docx [file mep-15-10821-s001.zip › _Educational Summary Report_10821.pdf]

MedEdPORTAL®
The Journal of Teaching and Learning Resources

Crossref

Original Publication

OPEN ACCESS

Constructing a Shared Mental Model for Feedback Conversations: Faculty Workshop Using Video Vignettes Developed by Residents

Alex Moroz, MD, MHPE*, Anna King, DO, Baruch Kim, DO, Heidi Fusco, MD, Kristin Carmody, MD, MHPE

*Corresponding author: alex.moroz@nyumc.org

Citation: Moroz A, King A, Kim B, Fusco H, Carmody K. Constructing a shared mental model for feedback conversations: faculty workshop using video vignettes developed by residents. *MedEdPORTAL*. 2019;15:10821. https://doi.org/10.15766/mep_2374-8265.10821

Copyright: © 2019 Moroz et al. This is an open-access article distributed under the terms of the Creative Commons Attribution license.

Appendices

A. Facilitator Guide.docx
B. Vignette Scripts.docx
C. Cocky Connor.mp4
D. Constructive Conversation .mp4
E. Defensive Debbie.mp4
F. Distracted Attending.mp4
G. Impersonal Attending.mp4
H. Self-Effacing Sammy.mp4
I. Session Evaluation.docx
J. Dimensions and Items.docx

All appendices are peer reviewed as integral parts of the Original Publication.

Abstract

Introduction: Providing feedback is a fundamental principle in medical education; however, as educators, our community lacks the necessary skills to give meaningful, impactful feedback to those under our supervision. By improving our feedback-giving skills, we provide concrete ways for trainees to optimize their performance, ultimately leading to better patient care. **Methods:** In this faculty development workshop, faculty groups used six feedback video vignettes scripted, enacted, and produced by residents to arrive at a shared mental model of feedback. During workshop development, we used qualitative analysis for faculty narratives combined with the findings from a focused literature review to define dimensions of feedback. **Results:** Twenty-three faculty (physical medicine and rehabilitation and neurology) participated in seven small-group workshops. Analysis of group discussion notes yielded 343 codes that were collapsed into 25 coding categories. After incorporating the results of a focused literature review, we identified 48 items grouped into 10 dimensions of feedback. Online session evaluation indicated that faculty members liked the workshop's format and thought they were better at providing feedback to residents as a result of the workshop. **Discussion:** Small faculty groups were able to develop a shared mental model of dimensions of feedback that was also grounded in medical education literature. The theme of specificity of feedback was prominent and echoed recent medical education research findings. Defining performance expectations for feedback providers in the form of a practical and psychometrically sound rubric can enhance reliable scoring of feedback performance assessments and should be the next step in our work.

Keywords

Feedback, Workshop, Faculty Development, Physical Medicine and Rehabilitation

Educational Objectives

By the end of this activity, learners will be able to:

1. Identify key behaviors in faculty-resident feedback conversations.
2. Describe dimensions of meaningful and impactful feedback.
3. Discuss effective strategies for managing challenging feedback conversations.

Introduction

The ultimate goal of assessment practices in health professional education is improved health care. High-quality, credible feedback is necessary for assessment to provide a meaningful mechanism through which physicians can be expected to grow[1] and provide better care. Feedback is fundamental to everything physicians do—it is an essential part of every framework, every curriculum, every teaching interaction.

When we explored the experiences of physical medicine and rehabilitation (PM&R) residents who were part of a structured program of self-assessment followed by faculty feedback,[2] we found that both residents and faculty receiving feedback felt they were not ready for the challenges encountered in real-life feedback conversations. The impetus for improving feedback-givers' skills was born out of the study

findings, as it turned out that learners were not easily fooled by substandard feedback.[3] Interestingly, a similar theme emerged from a mixed-methods study that focused on faculty receiving feedback from residents.[4] These combined findings across specialties and professional roles reinforced our belief that development of feedback-giving skills is an underexplored area of medical education delivery and scholarship, strengthening which may allow us to take another step towards improving physicians' performance and, ultimately, providing better patient care.[5]

Meaningful and impactful feedback conversations are not easy for most feedback providers. On the one hand, faculty members' tensions impact not only the feedback process—reaching the right balance of constructive and positive feedback, dealing with their own perceptions of self-efficacy, as well as the receptiveness, insight, potential, and skill of the residents—but also the resident-faculty relationship and contextual factors.[6] On the other hand, faculty from both university-based and community-based programs reported inadequate training and incomplete understanding of the best ways to deliver feedback[6] despite the availability of excellent practical guides.[7,8] This does not appear to be just a perception issue—a recent qualitative study of simulated feedback encounters suggested that faculty skills do not in fact match recommended practices in several areas.[9]

A number of curricula to improve faculty feedback-giving skills have been described by others, including several *MedEdPORTAL* publications. Some of these focus on peer or near-peer feedback. For example, Brown, Rangachari, and Melia developed an interactive multimodal feedback coaching workshop for residents giving feedback to interns.[10] Tews and colleagues created a course to improve resident skills in providing feedback to medical students.[11] Others have experimented with improving faculty feedback skills. For example, Schlair, Dyche, and Milan described a longitudinal faculty development program designed by faculty leaders and found positive impact on feedback quality as perceived by residents.[12] Sargeant and colleagues described a workshop based on a thoroughly researched[8,13-15] coaching model of feedback.[16]

Although the imposition of predeveloped frameworks and top-down faculty development is tempting and may be an efficient approach, the very process of creating the framework and developing a holistic shared mental model may be essential for learning and participant buy-in.[17] Our goal, therefore, was to develop a workshop that would allow faculty groups to construct a shared mental model that was locally grown and sensitive to specialty and institution, yet based on general principles supported by medical education.

This workshop adds to the existing body of work in one other way. Other scholars have identified variability in feedback recipients (four resident challenges) and suggested adjusting feedback provider approaches accordingly.[9] Since our workshop is based on scenarios that were selected, scripted, and enacted by feedback recipients (residents), it allowed us to also explore variability in feedback provider (faculty) behaviors. We believe that using more than one perspective in developing a shared mental model for feedback allowed us to highlight multiple facets of the feedback construct and understand it more fully.

Most importantly, in addition to promoting a deeper understanding of the development of the knowledge, attitudes, skills, and habits necessary for providing meaningful and impactful feedback, improving faculty feedback-giving skills may allow us to take another step towards improving physicians' performance and patient care.

Methods

Local Feedback Context

After surveying residents in our large (N = 36) PM&R program regarding feedback they had received[18] in 2015, we implemented a feedback bundle that included resident self-assessment, a faculty assessment, and a joint conversation. This process was facilitated by using an iPad app called PRIMES (locally developed based on the RIME [Reporter, Interpreter, Manager, Educator] framework,[19] with the addition of Professionalism and procedural Skills). Using their iPad, the residents first self-assessed (on their own)

across the PRIMES dimensions and created three learning goals. They then met with, and passed the device to, the faculty, who assessed the resident blindly, using the same framework. After faculty submission of their assessment, the app compared resident self-assessment and faculty assessment, resulting in visual highlighting of areas of agreement and disagreement. This served as a starting point for a feedback conversation. Each resident was required to engage in this process at least once a month and was encouraged to do so mid-rotation. This was reinforced during semiannual meetings with the program director and via emails from chief residents.

During 14 monthly rotations, 48 residents and 16 faculty members completed 343 PRIMES encounters. Each faculty member participated in a median of four encounters. Average resident compliance with the once-a-month requirement during the same time period was 71%, with most feedback encounters clustering during the last week of the rotation.

Development of Feedback Vignettes

A resident developed six vignette scripts based on some of the issues identified in our qualitative study[2]—lack of faculty engagement or time (Distracted Attending, Impersonal Attending), challenging resident behaviors (Cocky Connor, Defensive Debbie, Self-Effacing Sammy), and a portrayal of a constructive conversation from the resident perspective (Appendix B). The scripts were reviewed by one of us (Alex Moroz), who made minor edits. We subsequently recruited two residents (Baruch Kim, Anna King) and a faculty member (Heidi Fusco), who enacted the six vignettes. These were recorded and produced (Appendices C-H).

Workshop Implementation

We conducted this 1-hour workshop (in small groups) with faculty in the departments of PM&R and neurology who were feedback providers for our residents (using the PRIMES feedback bundle described above). For convenience, faculty at each clinical site (e.g., inpatient brain injury) or resident rotation (e.g., electrodiagnosis) formed distinct groups. All workshops were conducted by one of us (Alex Moroz), who took handwritten notes documenting the faculty discussion.

Development of a Shared Mental Model

To accomplish the goal of developing a shared mental model of useful feedback conversations, each group of faculty viewed the video vignettes and afterward had a discussion that focused on faculty behavior in the vignette, aiming to answer the following two questions: “What did faculty do well?” “What could faculty do better?”

Qualitative Analysis and Literature Review

Group responses were recorded as positive statements to distill strong feedback practices (e.g., “She did not maintain eye contact” became “maintaining eye contact”) and scanned to qualitative analysis software (ATLAS.ti V.1.5.3; Scientific Software Development GmbH, Berlin, Germany). Using segments of text identified in the notes, we defined coding categories and created a coding scheme that identified individual codes and their relationships. We reached consensus by discussing this scheme and individual coding categories.

In order to ensure both sensitivity to specialty and institution context and conformance to general principles supported by medical education theory, we conducted a focused literature review and used the results to group our items (codes) into broader dimensions of feedback.

Session Evaluation

Faculty participants received an online session-evaluation survey (three items, developed by the authors) focusing on the first two levels of Kirkpatrick’s hierarchy,[20] as well as suggestions for improving the workshop (Appendix I). After analyzing responses, the session evaluation was revised to include five questions and better align with educational objectives.

MedEdPORTAL®
The Journal of Teaching and Learning Resources

Materials/Logistics/Setup Needed for Implementation

In order to implement this workshop locally, the following materials and local resources are necessary:

- Buy-in and support from division head or department chair.
- Facilitator's guide (Appendix A).
- Faculty small-group facilitator and faculty champion (can be the same person).
- Support staff to schedule workshop and remind faculty.
- Sufficient space to accommodate small-group discussions.
- Working audiovisual equipment capable of playing video on a sufficiently large screen.

Results

Twenty-three faculty members participated in seven small-group workshops. Average group size was 3.3 (range: two to five participants), and there were 16 male and seven female faculty participants. Of the seven workshops, six were with PM&R faculty groups, and one was with a neurology faculty group.

Initial qualitative analysis of group discussion notes yielded 343 codes that were collapsed into 25 coding categories (Table). Each cell represents the number of times faculty (all groups) mentioned a particular coding category during discussion of the case in question.

Table. Distributions of Coding Categories Among the Six Vignettes

	Vignette						
**Coding Category**	**Cocky Connor**	**Constructive Conversation**	**Defensive Debbie**	**Distracted Attending**	**Impersonal Attending**	**Self-Effacing Sammy**	**Coding Category Total**
Being confident and staying in control	3	0	5	0	0	0	8
Being constructive without offending	6	2	1	3	1	1	14
Being honest about not enough facts or not enough time	0	0	1	1	5	0	7
Being organized and completing the encounter	1	0	1	1	0	1	4
Being polite and respectful	4	1	2	3	2	1	13
Being positive, using positive language	2	6	8	2	1	6	25
Being prepared	0	1	0	0	7	0	8
Being present, engaged and paying attention	1	0	2	8	5	3	19
Being specific and giving examples	6	17	15	4	1	8	51
Being warm, approachable, supportive, encouraging, reassuring	4	5	3	7	4	10	33
Confronting wrong perceptions and inappropriate behaviors	14	0	3	0	0	1	18
Dedicating time and minimizing disruptions	0	0	0	6	4	0	10
Defining expectations, reviewing performance over time	1	2	2	0	0	1	6
Discussing areas of improvement, action plan, and follow-up	4	8	1	0	0	1	14
Ensuring quiet, private, appropriate environment	3	0	0	1	0	1	5
Knowing the resident and basing feedback on objective facts	0	1	1	3	7	1	13
Listening and having a dialogue	1	2	1	0	0	4	8
Making eye contact and leaning forward	2	3	3	4	0	4	16
Not just "going through the motions"	0	0	0	4	0	0	4
Putting things in perspective	3	0	2	0	0	0	5
Reacting to resident answers, probing deeper	6	4	5	0	0	17	32
Redirecting and disarming	3	0	2	0	0	0	5
Referring up for wellness and psychiatric concerns	0	0	0	0	0	3	3
Starting with self-assessment	0	2	1	1	5	5	14
Staying calm, composed, and nonconfrontational	4	1	3	0	0	0	8
Vignette total	68	55	62	48	42	68	

After incorporating the results of a focused literature review, we identified 48 items that we grouped into 10 dimensions of feedback, supported by both our data and published literature (Figure; see Appendix J for the supporting literature references). Faculty groups identified 11 out of 32 items discussed in the literature (indicated by italics in the Figure).

Preparation, Engagement, Investment	•Being prepared<br>•Dedicating time; minimizing disruptions; ensuring quiet, private, appropriate environment<br>•Developing rapport (Roze des Ordons 2018, Sargeant 2015)<br>•Being present, engaged, paying attention<br>•Making eye contact and leaning forward<br>•Being honest about not enough time or not enough facts<br>•*Not just "going through the motions" (Moroz 2018)*<br>•Being organized and completing the encounter (Sargeant 2015)
Defining Expected Performance	•*Defining expectations for performance; clarifying expected standards (Johnson 2016)*<br>•Clarifying good performance (Nicol 2006); clarifying characteristics of good performance (Johnson 2016)<br>•Giving examples of good performance
Starting With Self-Assessment	•*Starting with self-assessment (Nicol 2006)*<br>•Encouraging honest self-assessment (Johnson 2016)<br>•Facilitating reflection and informed self-assessment (Ramani 2018)<br>•Exploring feedback recipient's perspective (Roze des Ordons 2018)
Beneficence, Encouragement, Respect	•*Being warm, approachable, supportive, encouraging (van de Ridder 2015), reassuring*<br>•Being positive and using positive language<br>•*Being polite and respectful (Johnson 2016, Sargeant 2015), nonjudgmental*<br>•Being constructive without offending<br>•Not threatening self-esteem (van de Ridder 2015); allowing to maintain self-concept (Eva 2012)<br>•Clear position of beneficence (Eva 2012)<br>•Creating positive learning climate (Ramani 2018)
Exploration, Reaction, Dialogue	•Listening<br>•Treating as a conversation (Lefroy 2015); meaningful conversation (Tekian 2017)<br>•*Exploring feedback recipient's perspective (Roze des Ordons 2018); probing deeper; elaborating content (van de Ridder 2015)*<br>•Reacting to feedback recipient's answers<br>•*Having a dialogue; encouraging a dialogue (Nicol 2006); encouraging recipient participation through dialogue (Johnson 2016)*
Using Observations and Facts	•Using individual physician data (Payne & Hysong 2016)<br>•Knowing the feedback recipient<br>•Basing feedback on observation (Lefroy 2015); basing feedback on regular observation (Tekian 2017); basing feedback on observed performance (Johnson 2016); using direct observation of performance to generate feedback data (Ramani 2018)<br>•Basing feedback on objective facts (Sargeant 2015)
Specificity and Using Examples	•*Being specific (van de Ridder 2015, Lefroy 2015)*<br>•*Giving examples; providing specific comments and examples (Johnson 2016, Sargeant 2015)*<br>•Specific and measurable feedback (Tekian 2017)<br>•Using specific strengths and weaknesses (Warm 2018); reviewing specific areas for improvement (Payne & Hysong 2016)
Confidence, Direction, Correction	•Staying calm, composed, nonconfrontational<br>•Redirecting and disarming<br>•Being confident and staying in control<br>•Confronting wrong perceptions and inappropriate behaviors<br>•Adapting directive approach for residents with insight gaps (Roze des Ordons 2018)
Individualizing the Conversation	•Diagnosing learner<br>•Adapting feedback to the specific needs of individual recipients (Roze des Ordons 2018)<br>•Tailoring feedback to be interpretable and palatable to the recipient (Eva 2012); tailoring feedback to individual trainee (Lefroy 2015)
Goals, Action Plan, Follow-up	•Setting goals (Johnson 2016, Sargeant 2015)<br>•*Discussing areas for improvement; specific areas for improvement (Payne & Hysong 2016)*<br>•Support for improvement strategies and scaffolding (Lefroy 2015)<br>•*Discussing action plan (Johnson 2016); actionable feedback (Lefroy 2015); planning for change (Tekian 2017)*<br>•Providing opportunities to close the gap (Nicol 2006)

Figure. Feedback dimensions and items identified by small faculty groups. Items in italics are those discussed in the published literature and also identified by the small faculty groups. See Appendix J for the published literature references.

The theme of specificity of feedback emerged as a prominent finding when we explored how the coding categories distributed across the feedback vignette scenarios (Table). Faculty agreed that the cocky resident needed confronting, the defensive resident needed specific examples, and the self-effacing resident needed exploration and support.

Four participants completed the online session-evaluation questionnaire (Appendix I). All reported that they liked (two) or liked a lot (two) the workshop's format and thought they were better at providing feedback to residents as a result of the workshop (four). Recommendations for improvement included providing more videos on good feedback technique, including an optimal feedback session, "keeping it up," and removing the titles of the videos as they might bias the viewers.

Discussion

In this faculty development workshop, small groups of faculty viewed feedback video vignettes together and arrived at a shared mental model of feedback. We used qualitative analysis for faculty narratives combined with the findings from a focused literature review to define dimensions of feedback and shared these with the participating faculty.

In a short time (1 hour), small faculty groups were able to develop a shared mental model of dimensions of meaningful and impactful feedback that was also grounded in medical education literature.

While individual group discussions varied, the characteristics of feedback that each group thought to be most valuable were similar across all groups.

Faculty groups identified 11 out of 32 items discussed in the published literature (Appendix J). The largest number of missed items clustered around the dimension of Starting With Self-Assessment, perhaps because the structured feedback encounter automatically included resident self-assessment and was taken for granted as part of the process by the participating faculty. Another explanation is that physicians, similar to other professions, do not accurately self-assess or include this process in their daily practice or cultural norms.[21] There were two dimensions (Preparation, Engagement, Investment and Confidence, Direction, Correction) that received more attention from the participating faculty than from the previously published literature. We hypothesize that this may have been a result of the specific scenarios selected for video vignettes. However, considering that these vignettes were created by residents and deemed representative of relevant feedback interactions, it is likely that these dimensions will be generalizable to other settings.

In fact, the theme of specificity of feedback not only appeared as one of the 10 dimensions (Individualizing the Conversation), it was also a prominent finding when we explored how the coding categories distributed across the feedback vignette scenarios (Table). Faculty agreed that the cocky resident needed confronting, the defensive resident needed specific examples, and the self-effacing resident needed exploration and support. This closely echoes the findings of Roze des Ordons, Cheng, Gaudet, Downar, and Lockyer,[22] who explored the challenges that faculty experienced and the approaches taken in adapting feedback conversations to different residents. While the faculty questioned their ability, they were able to adapt their approach to feedback, drawing on techniques of coaching for highly performing residents, directing for residents demonstrating insight gaps, mentoring and support for emotionally distressed residents, and mediation for overly confident residents.

Our work has several important limitations. As the workshop was conducted within a single institution and specialty, the items and dimensions identified may not be directly generalizable to other settings. Similarly, we decided not to change or remove the vignette titles as these represented our resident perceptions of the process. We think that including findings from a focused literature review may have mitigated this somewhat. In addition, feedback is a universal skill necessary across all specialties, and therefore, it would be expected that other settings would encounter similar challenges. Similarly, our work was limited to the

graduate medical education setting, and we do not know if our findings are generalizable to the contexts of undergraduate or postgraduate medical education or broader health professions education.

There were also challenges we encountered in our program evaluation approach. The response rate (four out of 23) was rather low, and in retrospect, we should have followed the initial survey invitation with several reminders (lesson learned). Additionally, our session-evaluation questionnaire focused on lower levels of Kirkpatrick's hierarchy, rather than on changes in faculty behaviors or, better yet, resident behavior changes or patient outcomes. While we acknowledge the rising difficulty of measuring outcomes with each step within the hierarchy, we also recognize the matching increase in the meaningfulness of the findings.

We believe that while these findings have deepened our theoretical understanding of the dimensions of feedback, defining performance expectations for feedback providers in the form of a practical and psychometrically sound rubric can increase reliability of scoring for feedback assessments and may be the next logical step in our work. Although rubrics may not ensure validity of judgment of feedback ratings per se, they can potentially promote learning and make teaching feedback providers easier by clarifying both the criteria and the expectations, thereby facilitating feedback and self-assessment.[23]

We offer the following recommendations to readers who might consider replicating this workshop. We do not think that a formal analysis of the participants' discussions, as was done here, is necessary after each workshop; on the other hand, explicit and frank discussion and local consensus-building process is paramount. By limiting the size of small groups to three to seven participants, the workshop can be scaled up to any number of faculty by manipulating the number of groups. We do not think that increasing group size is as effective because it would be difficult to insure engagement of all group members in larger groups. Finally, we found our workshop evaluation response rate to be very low with a single administration of the survey; we suggest at least two strategically timed reminders after the initial request for feedback.

Alex Moroz, MD, MHPE: Associate Professor, Department of Rehabilitation Medicine, New York University School of Medicine

Anna King, DO: Chief Resident, Department of Rehabilitation Medicine, New York University School of Medicine

Baruch Kim, DO: Chief Resident, Department of Rehabilitation Medicine, New York University School of Medicine

Heidi Fusco, MD: Clinical Assistant Professor, Department of Rehabilitation Medicine, New York University School of Medicine

Kristin Carmody, MD, MHPE: Associate Professor, Department of Emergency Medicine, New York University School of Medicine

Disclosures
None to report.

Funding/Support
None to report.

Ethical Approval
Reported as not applicable.

References

1. Eva KW, Armson H, Holmboe E, et al. Factors influencing responsiveness to feedback: on the interplay between fear, confidence, and reasoning processes. *Adv Heal Sci Educ Theory Pract*. 2012;17(1):15-26. https://doi.org/10.1007/s10459-011-9290-7
2. Moroz A, Horlick M, Mandalaywala N, Stern DT. Faculty feedback that begins with resident self-assessment: motivation is the key to success. *Med Educ*. 2018;52(3):314-323. https://doi.org/10.1111/medu.13484
3. LaDonna KA, Watling C. In search of meaningful feedback conversations. *Med Educ*. 2018;52(3):250-251. https://doi.org/10.1111/medu.13518

4. Carmody K, Walia I, Coneybeare D, Kalet A. Can a leopard change its spots? A mixed methods study exploring emergency medicine faculty perceptions of feedback, strategies for coping and barriers to change [master's thesis]. Maastricht, the Netherlands: Maastricht University School of Health Education; 2017.
5. Moroz A, Carmody K. Thank you for your feedback? An imperative to improve feedback givers' skills. *MedEdPublish*. 2018;7(1):51. https://doi.org/10.15694/mep.2018.0000051.1
6. Kogan JR, Conforti LN, Bernabeo EC, Durning SJ, Hauer KE, Holmboe ES. Faculty staff perceptions of feedback to residents after direct observation of clinical skills. *Med Educ.* 2012;46(2):201-215. https://doi.org/10.1111/j.1365-2923.2011.04137.x
7. Lefroy J, Watling C, Teunissen PW, Brand P. Guidelines: the do's, don'ts and don't knows of feedback for clinical education. *Perspect Med Educ.* 2015;4(6):284-299. https://doi.org/10.1007/s40037-015-0231-7
8. Sargeant J, Lockyer JM, Mann K, et al. The R2C2 model in residency education: how does it foster coaching and promote feedback use? *Acad Med*. 2018:93(7):1055-1063. https://doi.org/10.1097/ACM.0000000000002131
9. Roze des Ordons AL, Cheng A, Gaudet JE, Downar J, Lockyer JM; Cumming School of Medicine Feedback and Debriefing Investigators. Exploring faculty approaches to feedback in the simulated setting: are they evidence informed? *Simul Healthc.* 2018;13(3):195-200. https://doi.org/10.1097/SIH.0000000000000289
10. Brown LE, Rangachari D, Melia M. Beyond the sandwich: from feedback to clinical coaching for residents as teachers. *MedEdPORTAL*. 2017;13:10627. https://doi.org/15766/mep_2374-8265.10627
11. Tews M, Quinn-Leering K, Fox C, Simonson J, Ellinas E, Lemen P. Residents as educators: giving feedback. *MedEdPORTAL*. 2014;10:9658. https://doi.org/10.15766/mep_2374-8265.9658
12. Schlair S, Dyche L, Milan F. Longitudinal faculty development program to promote effective observation and feedback skills in direct clinical observation. *MedEdPORTAL*. 2017;13:10648. https://doi.org/10.15766/mep_2374-8265.10648
13. Sargeant J, Lockyer J, Mann K, et al. Facilitated reflective performance feedback: developing an evidence- and theory-based model that builds relationship, explores reactions and content, and coaches for performance change (R2C2). *Acad Med*. 2015;90(12):1698-1706. https://doi.org/10.1097/ACM.0000000000000809
14. Sargeant J, Eva KW, Armson H, et al. Features of assessment learners use to make informed self-assessments of clinical performance. *Med Educ.* 2011;45(6):636-647. https://doi.org/10.1111/j.1365-2923.2010.03888.x
15. Sargeant J, Mann K, Manos S, et al. R2C2 in action: testing an evidence-based model to facilitate feedback and coaching in residency. *J Grad Med Educ.* 2017;9(2):165-170. https://doi.org/10.4300/JGME-D-16-00398.1
16. Sargeant J, Armson H, Driessen E, et al. Evidence-informed facilitated feedback: the R2C2 feedback model. *MedEdPORTAL*. 2016:12:10387. https://doi.org/10.15766/mep_2374-8265.10387
17. Kogan JR, Conforti LN, Bernabeo E, Iobst W, Holmboe E. How faculty members experience workplace-based assessment rater training: a qualitative study. *Med Educ.* 2015;49(7):692-708. https://doi.org/10.1111/medu.12733
18. Yarris LM, Linden JA, Hern HG, et al; for Emergency Medicine Education Research Group (EMERGe). Attending and resident satisfaction with feedback in the emergency department. *Acad Emerg Med*. 2009;16(suppl 2):S76-S81. https://doi.org/10.1111/j.1553-2712.2009.00592.x
19. Rodriguez RG, Pangaro LN. AM Last Page: mapping the ACGME competencies to the RIME framework. *Acad Med*. 2012;87(12):1781. https://doi.org/10.1097/ACM.0b013e318271eb61
20. Kirkpatrick DL. *Evaluating Training Programs: The Four Levels*. San Francisco, CA: Berrett-Koehler; 1994.
21. Davis DA, Mazmanian PE, Fordis M, Van Harrison R, Thorpe KE, Perrier L. Accuracy of physician self-assessment compared with observed measures of competence: a systematic review. *JAMA*. 2006;296(9):1094-1102. https://doi.org/10.1001/jama.296.9.1094
22. Roze des Ordons A, Cheng A, Gaudet J, Downar J, Lockyer J. Adapting feedback to individual residents: an examination of preceptor challenges and approaches. *J Grad Med Educ.* 2018;10(2):168-175. https://doi.org/10.4300/JGME-D-17-00590.1
23. Jonsson A, Svingby G. The use of scoring rubrics: reliability, validity and educational consequences. *Educ Res Rev.* 2007;2(2):130-144. https://doi.org/10.1016/j.edurev.2007.05.002

Received: March 23, 2019 | **Accepted:** November 12, 2018 | **Published:** May 1, 2019
