# Supplementary material for: Constructing a Shared Mental Model for Feedback Conversations: Faculty Workshop Using Video Vignettes Developed by Residents
Source: MedEdPORTAL. 2019 May 1;15:10821. doi: 10.15766/mep_2374-8265.10821 (PMC6519682; doi:10.15766/mep_2374-8265.10821)
Supplement: Supplementary file 1 — A. Facilitator Guide.docx B. Vignette Scripts.docx C. Cocky Connor.mp4 D. Constructive Conversation.mp4 E. Defensive Debbie.mp4 F. Distracted Attending.mp4 G. Impersonal Attending.mp4 H. Self-Effacing Sammy.mp4 I. Session Evaluation.docx J. Dimensions and Items.docx [file mep-15-10821-s001.zip › A. Facilitator Guide.docx]

**Workshop goals**

By the end of this activity, faculty will be able to:

1. Identify key behaviors in faculty-resident feedback conversations
2. Describe dimensions of meaningful and impactful feedback
3. Discuss effective strategies for managing challenging feedback conversations

**Preparation**

1. Ensure buy-in from your division head or department chair
2. Select dates, places, support staff
3. Invite small groups of faculty for whom feedback conversations are most relevant; depending on your needs, these may be faculty who give most or least, best or worst feedback. We found that groups including between 3 and 7 people tend to work best. How will you organize and mix the groups?
4. Test audiovisual equipment in the location of the workshop to ensure both audio and video are working smoothly
5. Decide on and provide means of recording faculty discussion/consensus. Who will be recording? Will this be an audio recording with subsequent transcription, hand-written notes (paper or tablet), typed notes? Where/how will you store the data for each small group?
6. Who will be facilitating? Do they need training?

**Facilitation**

1. Welcome everyone and introduce the workshop goals (listed above)
2. Have the group view the video vignettes, one at a time. Ask if the group would like to see it again before discussing.
3. After viewing each vignette, pause and facilitate a discussion focused on faculty behavior in the vignette.
4. Ask the faculty, as a group, to answer the following two questions: “what did faculty in the video do well?”, and “what could faculty in the video do better?”
5. If group members are struggling to come up with answers, use the following alternative prompts: “if you were there, what could you do differently?”, or “what do you think worked while providing feedback to this resident?”, or “what did not work in this scenario?”
6. Use group discussion to resolve any disagreements or lack of clarity.
7. Record the key points of agreement using your preferred method.
8. Repeat the steps 3-6 for the remaining vignettes.
9. Store the data for future use.

**Follow up**

1. After each small group workshop, send out a Session Evaluation (*Appendix I*) by email. We suggest at least 2 strategically timed reminders after the initial request in order to improve response rate.
2. After conducting all small group workshops, review and summarize the key points of agreement for each group.
3. Share your findings in a live general faculty presentation.
4. End the meeting with agreeing and committing to specific next steps for improving feedback quality.
5. Follow up the live meeting with an email summary.
6. Analyze responses to Session Evaluation survey, and modify your next workshop as needed.
