# Supplementary material for: Constructing a Shared Mental Model for Feedback Conversations: Faculty Workshop Using Video Vignettes Developed by Residents
Source: MedEdPORTAL. 2019 May 1;15:10821. doi: 10.15766/mep_2374-8265.10821 (PMC6519682; doi:10.15766/mep_2374-8265.10821)
Supplement: Supplementary file 1 — A. Facilitator Guide.docx B. Vignette Scripts.docx C. Cocky Connor.mp4 D. Constructive Conversation.mp4 E. Defensive Debbie.mp4 F. Distracted Attending.mp4 G. Impersonal Attending.mp4 H. Self-Effacing Sammy.mp4 I. Session Evaluation.docx J. Dimensions and Items.docx [file mep-15-10821-s001.zip › B. Vignette Scripts.docx]

**Faculty-resident feedback scenarios:**

1. Defensive Debbie (resident variation)
2. Self-Effacing Sammy (resident variation)
3. Cocky Connor (resident variation)
4. “ADHD”: Distracted Attending (attending variation)
5. “Cheap Date”: Impersonal Attending (attending variation)
6. Constructive Conversation

**Scenario 1: Defensive Debbie**

Description:

Attending feeling very uncomfortable providing feedback to a defensive resident.

Teaching Points/Key Aspects of Feedback:

being specific and giving examples; being positive and using positive language; being confident and staying in control

Attending: (Thinking out loud.) *Oh, brother . Here comes Debbie. How did Dr. Im get out of this one again?*

Debbie: Hi, Dr. Fusco. Thanks for doing my Primes.

Attending: Of course. It’s my pleasure! (with exaggerated smile.)

Debbie: (Debbie presenting Dr. Fusco with iPad.)

Attending: Now let’s see…. (Attending gliding through the format, then pausing at the descriptor before typing her response. Then hitting submit with a self-satisfied nod of her head.)

Attending: Ok. So, overall, I think you’re doing fine. Naturally, there’s always room for improvement….

Debbie: (Nervously smiling.) Oh, yeah? Like what?

Attending: (Pausing before continuing, remembering how Debbie can be.) Well , I just meant that in general….

Debbie: (Quickly interjecting.) No. Like, specifically… how??

Attending: Riight…. Just so you know, Professionalism , Reporting… you’re strong.

Debbie: Uh huh. (Nodding. Sitting on edge of seat for more feedback.)

Attending: (Hesitating in anticipation of Debbie’s reaction.) For Interpretation and Management, I gave you Competent.

Debbie: Umm. Ok. Umm…. Can I ask why?

Attending: Well, Debbie. Again, you’re doing fine. But you’re still learning. You’re exactly where you’re expected to be at your level.

Debbie: Umm. Yeah. No. Fine. (Awkward silence) I mean, I know I’m better than (fill in the blank name), but fine.

Attending: You do realize that this is nothing negative. And it’s not even something going into your personal file….

Debbie: There’s a personal file on me?!

Attending: Never mind.

**Scenario 2: Self-Effacing Sammy**

Description:

Resident is resisting attending’s feedback to invalidate attending’s negative opinion of her performance.

Teaching Points/Key Aspects of Feedback:

Reacting to resident answers, probing deeper; being warm, approachable, supportive, encouraging, reassuring; being specific and giving examples

Sammy: Hi, Dr. Fusco. Is now an ok time to do my Primes?

Attending: Hi, Sammy. Of course. Not a problem.

Sammy: I realize you’re busy. We can do it another time. I’m sorry….

Attending: (Smiling) For the 5^th^ time, it’s ok. Take a seat. Where’s your iPad?

Sammy: (Pulling out her iPad.)

Attending: Did you submit your responses?

Sammy: No. Not yet. Do you want to see my responses?

Attending: That’s ok. We can go over that later.

Sammy: (Uncomfortably hitting send. Then handing over her iPad.)

Attending: (Cheerily making her selections and typing her response. Then hitting submit.) Ok, Sammy. I have to say, you’re doing a great job.

Sammy: You don’t have to say that.

Attending: No, I mean it. You’re taking more ownership of the patients. You’re working great with the team.

Sammy: Yeah, but I’m still not doing a great job teaching.

Attending: Sammy, that’ll come with time and confidence.

Sammy: I know! I’m not confident.

Attending: (Amused and shaking her head.) Well, we all have things we need to work on. But, overall, you are managing the patients well.

Sammy: I guess.

Attending: Your Interpretation is solid.

Sammy: Yeah, but it could be better.

Attending: Again, that’s something that will come with experience. But, overall, you’re doing great.

Sammy: (Looking seemingly unsatisfied.) If you say so.

Attending: (Awkward silence.)

**Scenario 3: Cocky Connor**

Description:

Attending is providing feedback to an overly confident and arrogant resident.

Teaching Points/Key Aspects of Feedback:

confronting wrong perceptions and inappropriate behaviors

Connor: Hey, Dr. Fusco.

Attending: Hi, Connor. How are you? (Looking at her watch.)

Connor: Great! I just got off the phone with the fellowship director at HSS.

Attending: (Unfazed.) That’s nice.

Connor: Yeah. Even though I’m a PGY-2, it’s good to get it in early if you’re gunning for pain.

Attending: Gotcha.

Connor: (Pulling out his iPad and handing it over to the attending.)

Attending: Thank you.

Connor: No problem.

Attending: Now, let’s see…. (Continues to make her selections and type her response. Then hit submit.) Ok, Connor. So, you continue to do a great job with Reporting, Interpretation, and Management.

Connor: (Smug look on face.) I know.

Attending: (Looking a little put off.) And we haven’t had many procedures this rotation.

Connor: No worries. I’ve got those covered. Been practicing after hours.

Attending: (With quizzical look on her face.) Now, in terms of Professionalism, that could use a little improving.

Connor: (Chortling) You’re kidding, right?

Attending: I understand you’ve been having a few issues with nursing. And the therapists say you’ve been a bit dismissive and unresponsive to their requests.

Connor: Dr. Fusco. Come on! You know how idiotic those nurses can be. And, I’m not gonna drop everything just because some therapist says a patient is dizzy. I mean, deal with it. Give the patient some water.

Attending: Yeah, that’s kind of what I mean. Connor, Physiatry is unique in that we work as a collaborative.

Connor: Well, I’m the doctor.

Attending: Well, you are the resident. But, regardless, we have to work as a team and foster a respectful environment. Everyone brings their own experiences and skills to the table.

Connor: Come on, some of the nurses don’t even read the orders. And,….

Attending: Connor, I realize sometimes it can be frustrating but you have to find a way to work better with the team. It will help you out in the long run.

Connor: Yeah, well that’s why I’m doing pain. I won’t have to deal with this bs.

Attending: (Deflated.) Sure, Connor.

ALTERNATIVE POSSIBILITY:

Attending: (In a friendly tone.) Procedural skills can use some improving. But, that’s why you’re in residency.

Connor: Ok. But, come on. Not to name names but you’ve got to admit I’m better than most other residents in our program.

Attending: This is not about comparing you to someone else.

Connor: Yeah But, for real. You do agree that I’m in the top percentile of my class, right?

**Scenario 4: “ADHD”: Distracted Attending**

Description:

Resident sits in front of attending while politely waiting for her to deal with looking up some things on computer. Then, during interaction, attending keeps allowing in various distractions: looks at computer from time to time, cell phone going off, she invites floor resident into room and have him wait while finishing up interaction, etc.

Teaching Points/Key Aspects of Feedback:

being present, engaged, and paying attention; being warm, approachable, supportive, encouraging, reassuring

Resident: Hi, Dr. Fusco. Is now a good time?

Attending: Hey, Amir. Umm. Yeah. No. Yeah. Yes. Sit down.

Resident: (Tentatively) Ok. So I already submitted my section. (Handing over iPad to attending.)

Attending: Ok. (Sending a text from her cell phone.)

Resident: Feel free to take your time.

Attending: What? No. Ok. We’re good. Now let me see…. (Proceeds to make selections. Gets phone call.) Oh, hey. Sorry. Just one sec. (Proceeds to take call.)

Resident: (Politely waiting.) Do you want me to step out?

Attending: (Nodding “No” and mouthing, “Just one sec.”) Uh, huh. Yeah. Sure. I’ll do that last consult. Umm. I’ll be there in 10 minutes. (Hangs up.) Sorry. Where were we?

Resident: Seriously, we can do this tomorrow or some other time.

Attending: No, no. Let’s just get this over with.

Resident: (Looking a little uncomfortable.)

Attending: Ok. Strong, strong, competent.

(Here’s knock on door.)

Attending: Come in.

Resident 2: Hi, Dr. Fusco. You wanted to go over your list? Oh, hey, Amir.

Attending: Yes. Just give me a second. Take a seat.

Resident 2: Ok.

Resident 1: (Feeling awkward.) Are you sure you don’t want to just wait.

Attending: (Disregarding comment. After submitting feedback.) Voila! Ok, Amir. You’re doing better. But you still need to work on your knowledge base.

Resident 2: (Nodding.)

Resident 1: (Look of dread and just wanting the experience to be over with asap.) Ok. Thanks. Are we done?

Attending: Yeah. I’ve got to be up on the ward in 10 minutes.

**Scenario 5: “Cheap Date”: Impersonal Attending**

Description:

Attending brusquely provides feedback to resident she barely knows.

Teaching Points/Key Aspects of Feedback:

being prepared; knowing the resident and basing feedback on objective facts

Attending: Ok. So where’s your iPad?

Resident: Here you go. (Handing over iPad.)

Attending: (With stern look on face, goes through the selections and types response. Then submits feedback and closes iPad.) So, realize we’ve worked together maybe three times?

Resident: I think I sat in on one of your cases.

Attending: Fine. Anyways. In all that time, I’ve gotten an overall sense of how you are as a resident.

Resident: Ok.

Attending: How do you feel you did?

Resident: Well, I know there’s room for improvement, but I gave myself Competent….

Attending: (Cutting off resident.) Yeah, no. Ok. Listen, Tom

Resident: Bob

Attending: Right. Bob? You’re in residency for a reason. Hopefully, in time, you’ll improve. But for now I have to say you’re at a beginner’s level. You’re a PGY-2?

Resident: 3

Attending: (Wincing.) Ooh.

Resident: What?

Attending: No. Nothing. You still have time. Anyways, I have to get back upstairs.

Resident: (Perplexed and demoralized.) Ok.

**Scenario 6: Constructive Conversation**

Description:

Resident and attending go through the feedback encounter in a constructive way.

Teaching Points/Key Aspects of Feedback:

being specific and giving examples; discussing areas of improvement, action plan, and follow-up

Attending: Ok. So how do you think you’re doing?

Resident: Well, I think I’m doing ok. I definitely think I’ve become more efficient. I’m starting to feel a little more comfortable managing the other residents. But, I think I still need to work on my knowledge base. I mean I try to read whenever I get a chance, but I’m “not there” yet.

Attending: Hmm. Well that was insightful. In general, you’re doing a good job. I agree with your self-assessment for the most part. I see for professionalism you gave yourself beginning. Actually, I put competent.

Resident: Oh. Ok.

Attending: But, I agree with your assessment. See what I wrote? (Turning iPad for resident to read.)

Resident: (Reading out loud.) “Needs to work on knowledge base.”

Attending: It’s just something we all have to work on.

Resident: I know, I know. But, it’s so hard to find the time.

Attending: Believe me, I understand. But, even if you read something or do some questions for just 15 minutes a day. It all adds up. You can even read up on your patients. That really helps make the material stick. The more you do so the better you’ll get at interpreting your findings and the more confident you’ll feel managing your juniors.

Resident: Yeah. I can see that.

Attending: Overall, I’ve seen the improvement. And you are such a hard worker. We all see that . But this will just help you take you to that next level.

Resident: That makes sense. Thanks, Dr. Fusco.
