# Supplementary material for: Constructing a Shared Mental Model for Feedback Conversations: Faculty Workshop Using Video Vignettes Developed by Residents
Source: MedEdPORTAL. 2019 May 1;15:10821. doi: 10.15766/mep_2374-8265.10821 (PMC6519682; doi:10.15766/mep_2374-8265.10821)
Supplement: Supplementary file 1 — A. Facilitator Guide.docx B. Vignette Scripts.docx C. Cocky Connor.mp4 D. Constructive Conversation.mp4 E. Defensive Debbie.mp4 F. Distracted Attending.mp4 G. Impersonal Attending.mp4 H. Self-Effacing Sammy.mp4 I. Session Evaluation.docx J. Dimensions and Items.docx [file mep-15-10821-s001.zip › I. Session Evaluation.docx]

**Appendix C. Session evaluation**

Dear Faculty:

Thank you for participating in our feedback skill development group session! Your contribution is invaluable in helping our residents grow as clinicians and ultimately improving the care they provide.

Please take a minute to answer these three questions, so that we can make this session even better next time:

**Question 1. How did this format work for you?**

I liked it a lot

I liked it

I am neutral

I did not like it

I did not like it at all

**Question 2. How did this session impact your ability to provide feedback to residents?**

I am much better at being able to provide feedback to residents

I am better at being able to provide feedback to residents

I am about the same

My ability to provide feedback to residents is less

My ability to provide feedback to residents is much less

**Question 3. Please list 3 key behaviors/dimensions for every faculty-resident feedback conversation**

**­­­­­­­**_______________________________________________

_______________________________________________

_______________________________________________

**Question 4. Please list 3 potential strategies for managing challenging feedback conversations**

_______________________________________________

_______________________________________________

_______________________________________________

**Question 5. How can we improve this session?**
