# Supplementary material for: Constructing a Shared Mental Model for Feedback Conversations: Faculty Workshop Using Video Vignettes Developed by Residents
Source: MedEdPORTAL. 2019 May 1;15:10821. doi: 10.15766/mep_2374-8265.10821 (PMC6519682; doi:10.15766/mep_2374-8265.10821)
Supplement: Supplementary file 1 — A. Facilitator Guide.docx B. Vignette Scripts.docx C. Cocky Connor.mp4 D. Constructive Conversation.mp4 E. Defensive Debbie.mp4 F. Distracted Attending.mp4 G. Impersonal Attending.mp4 H. Self-Effacing Sammy.mp4 I. Session Evaluation.docx J. Dimensions and Items.docx [file mep-15-10821-s001.zip › J. Dimensions and Items.docx]

Feedback Dimensions and Items identified by small faculty groups.

Preparation, Engagement, Investment

- Being prepared
- Dedicating time; minimizing disruptions; ensuring quiet, private, appropriate environment
- Developing rapport (1,2)
- Being present, engaged, paying attention
- Making eye contact and leaning forward
- Being honest about not enough time or not enough facts
- Not just ‘going through the motionsʼ (3)
- Being organized and completing the encounter (2)

Defining Expected Performance

- Defining expectations for performance; clarifying expected standards (4)
- Clarifying good performance (5); clarifying characteristics of good performance (4)
- Giving examples of good performance

Starting with Self-Assessment

- Starting with self-assessment (5)
- Encouraging honest self-assessment (4)
- Facilitating reflection and informed self-assessment (6)
- Exploring feedback recipient's perspective (1)

Beneficence, Encouragement, Respect

- Being warm, approachable, supportive, encouraging (7), reassuring
- Being positive and using positive language
- Being polite and respectful (2,4), non-judgemental
- Being constructive without offending
- Not threatening self-esteem (7); allowing to maintain self-concept (8)
- Clear position of beneficence (8)
- Creating positive learning climate (6)

Exploration, Reaction, Dialogue

- Listening
- Treating as a conversation (9); meaningful conversation (10)
- Exploring feedback recipient's perspective (1); probing deeper; elaborating content (7)
- Reacting to feedback recipient's answers
- Having a dialogue; encouraging a dialogue (5); encouraging recipient participation through dialogue (4)

Using Observations and Facts

- Using individual physician data (11)
- Knowing the feedback recipient
- Basing feedback on observation (9); basing feedback on regular observation (10); basing feedback on observed performance (4); using direct observation of performance to generate feedback data (6)
- Basing feedback on objective facts (2)

Specificity and using Examples

- Being specific (7,9)
- Giving examples; providing specific comments and examples (2,4)
- Specific and measurable feedback (10)
- Using specific strengths and weaknesses (12); reviewing specific areas for improvement (11)

Confidence, Direction, Correction

- Staying calm, composed, nonconfrontational
- Redirecting and disarming
- Being confident and staying in control
- Confronting wrong perceptions and inappropriate behaviors
- Adapting directive approach for residents with insight gaps (13)

Individualizing the Conversation

- Diagnosing learner
- Adapting feedback to the specific needs of individual recipients (13)
- Tailoring feedback to be interpretable and palatable to the recipient (8); tailoring feedback to individual trainee (9)

Goals, Action Plan, Follow-Up

- Setting goals (2,4)
- Discussing areas for improvement; specific areas for improvement (11)
- Support for improvement strategies and scaffolding (9)
- Discussing action plan (4); actionable feedback (9); planning for change (10)
- Providing opportunities to close the gap (5)

**References**

1. Roze Des Ordons A, Cheng A, Gaudet J, Downar J, Lockyer J. Adapting Feedback to Individual Residents: An Examination of Preceptor Challenges and Approaches. J Grad Med Educ [Internet]. 2018;April. Available from: http://www.jgme.org/doi/pdf/10.4300/JGME-D-17-00590.1

2. Sargeant J, Lockyer J, Mann K, Holmboe E, Silver I, Armson H, et al. Facilitated reflective performance feedback: developing an evidence- and theory-based model that builds relationship, explores reasctions and content, and coaches for performance change (R2C2). Acad Med [Internet]. 2015 Dec [cited 2016 Jul 22];90(12):1698–706. Available from: http://www.ncbi.nlm.nih.gov/pubmed/26200584

3. Moroz A, Horlick M, Mandalaywala N, Stern DT. Faculty feedback that begins with resident self-assessment: motivation is the key to success. Med Educ [Internet]. 2018 Dec 4 [cited 2017 Dec 5];52(3):314–23. Available from: http://doi.wiley.com/10.1111/medu.13484

4. Johnson CE, Keating JL, Boud DJ, Dalton M, Kiegaldie D, Hay M, et al. Identifying educator behaviours for high quality verbal feedback in health professions education: literature review and expert refinement. BMC Med Educ [Internet]. 2016;16(1):96. Available from: http://www.biomedcentral.com/1472-6920/16/96

5. Nicol D, MacFarlane-Dick D. Formative assessment and selfregulated learning: A model and seven principles of good feedback practice. Stud High Educ. 2006;31(2):199–218.

6. Ramani S, Könings KD, Mann K V., Pisarski EE, van der Vleuten CPM. About Politeness, Face, and Feedback. Acad Med [Internet]. 2018;1. Available from: http://insights.ovid.com/crossref?an=00001888-900000000-97959

7. van de Ridder JMM, Mcgaghie WC, Stokking KM, ten Cate OTJ. Variables that affect the process and outcome of feedback, relevant for medical training: A meta-review. Med Educ. 2015;49(7):658–73.

8. Eva KW, Armson H, Holmboe E, Lockyer J, Loney E, Mann K, et al. Factors influencing responsiveness to feedback: On the interplay between fear, confidence, and reasoning processes. Adv Heal Sci Educ. 2012;17(1):15–26.

9. Lefroy J, Watling C, Teunissen PW, Brand P. Guidelines: the do’s, don’ts and don’t knows of feedback for clinical education. Perspect Med Educ [Internet]. 2015;4(6):284–99. Available from: http://www.pubmedcentral.nih.gov/articlerender.fcgi?artid=4673072&tool=pmcentrez&rendertype=abstract

10. Tekian A, Watling CJ, Roberts TE, Steinert Y, Norcini J. Qualitative and quantitative feedback in the context of competency-based education. Med Teach. 2017;39(12):1245–9.

11. Payne VL, Hysong SSJ, Hattie J, Timperley H, Hysong SSJ, Hysong SSJ, et al. Model depicting aspects of audit and feedback that impact physicians’ acceptance of clinical performance feedback. BMC Health Serv Res [Internet]. 2016;16(1):260. Available from: http://bmchealthservres.biomedcentral.com/articles/10.1186/s12913-016-1486-3

12. Warm E, Kelleher M, Kinnear B, Sall D. Feedback on Feedback as a Faculty Development Tool. JGME. 2018;June:354–5.

13. Roze des Ordons AL, Cheng A, Gaudet JE, Downar J, Lockyer JM. Exploring Faculty Approaches to Feedback in the Simulated Setting. Simul Healthc J Soc Simul Healthc [Internet]. 2018;00(00):1. Available from: http://insights.ovid.com/crossref?an=01266021-900000000-99602
